# Evaluating the Practice of Preventive Behaviors and the Fear of COVID-19 among Dentists in Oradea Metropolitan Area after the First Wave of Pandemic; a Cross-Sectional Study

**DOI:** 10.3390/healthcare9040443

**Published:** 2021-04-09

**Authors:** Raluca Iurcov, Lavinia Maria Pop, Gabriela Ciavoi, Magdalena Iorga

**Affiliations:** 1 Dental Medicine Department, Faculty of Medicine and Pharmacy Oradea, University of Oradea, 410073 Oradea, Romania; riurcov@uoradea.ro (R.I.); gciavoi@uoradea.ro (G.C.); 2 Faculty of Psychology and Education Sciences, “Alexandru Ioan Cuza” University, 700554 Iasi, Romania; lavinia.pop@student.uaic.ro; 3 Behavioural Sciences Department, “Grigore T. Popa” University of Medicine and Pharmacy, 700115 Iasi, Romania

**Keywords:** healthcare, dentistry, COVID 19, pandemic, fear of COVID 19, preventive behaviors, clinical practice, stress

## Abstract

Dental clinics were suspected to be a hotspot for nosocomial transmission of COVID-19 due to the easy spread of the virus. The study investigated the preventive behaviors applied in dentistry settings and the level of fear of COVID-19 infection among dentists. A total of 83 respondents (34.94% male and 63.86% female) were included in the research. Sociodemographic data were collected, together with new institutional and personal rules regarding preventive behaviors. Fear of COVID-19 Scale was used to measure the fear of infection. Data was analyzed using SPSS (v.25, SPSS Inc., Chicago, IL, USA). During the first seven months of confinement, 3.9% of dentists were confirmed with COVID-19 and one fourth treated confirmed positive patients. A quarter of the doctors declared that they had periods when they lived away from home being afraid of transmitting the disease to their family members, and significant data were found in doctors being parents. The closure of dental offices had a negative impact on the financial situation of dentists, especially on those working in rural area offices. Many doctors encountered difficulties in purchasing protective suits and medical supplies, and more than half of the respondents (65.1%, *N* = 54) focused on the quality of protective suits when purchasing them. More than half of the dentists were trained how to use them. The score for fear of COVID 19 was similar to dentists from other countries. Respondents with chronic diseases were more prone to show higher level of anxiety when following the news and stories related to COVID-19 on TV, media, or social networks. One third of dentists mentioned that they had treated exclusively specific urgent dental problems since the onset of the pandemic and more than 13.3% declared that they refused to provide medical assistance to some specific pathologies because of the fear of infection. The results reflect new challenges and rules adopted by dentists in order to diminish the risk of infection and the impact of pandemic considering their psychological, familial, and financial context. Policymakers and professional associations around may benefit from these findings while formulating guidelines to support dentists during COVID-19 or any future pandemics.

## 1. Introduction

On 11 March 2020, the World Health Organization (WHO) declared that the COVID-19 situation can be qualified as a global pandemic because of its fast spread worldwide, with 100 countries affected at that time [1]. With the establishment of the state of emergency by Presidential Decree, on 16 March, a lot of measures were taken to diminish the risk of infection with SARS-CoV-2 (closing schools and universities, markets, and large malls, recommending tele-working for those employed in not vital sectors, imposing strict rules for social distancing, wearing medical protective masks, etc.). The first positive patient was confirmed in Romania on 26 February 2020 (direct contact with an Italian citizen). In Oradea, patient-zero was confirmed on 13 March 2020 (a female patient returned from Italy). Taking into consideration the increasing number of returning citizens, especially from European countries considered “red zones” and the general situation in Europe, a total lockdown was imposed on 21 March 2020. Starting with this date, dental offices had to close due to the high risk of infection and only public emergency offices continued to be open with but providing a limited number of procedures. A total lockdown was imposed during 21 March and 15 May, being the single one from the beginning of the pandemic until now. The number of public dental offices in Romania represents five percent of the total (and these are either school, student, or emergency offices). So, some private dental offices could sustain emergency services only if they met the rules for preventing infection legally regulated by law. The number of such dental offices was extremely low because it was very difficult to respect the preventive rules (impossible to procure or very expensive prices for additional protective equipment, special disinfection devices, disinfectants, etc.). Starting with the end of lockdown, dental offices could open. In the Romanian medical sector, only hospital and emergency units continued to provide medical care for those affected by the infection with SARS CoV-2 or to patients in emergency state. By Military Ordinance, common medical care was restricted, and public hospitals were categorized by providing/non-providing COVID-19 medical support [2]. Consequently, dental clinics and dental radiology offices were closed, and dentists provided solely emergency care. For these ones, The National Executive Office of the Romanian College of Dentists established regulations for the prevention of spread of the disease and some guidelines for providing dental care in case of dental emergency [3]. The following pathologies were considered emergencies: post-extractional hemorrhage; pain due to acute pulpitis; pain due to acute apical periodontitis; pericoronitis of the impacted teeth; post-extractional alveolitis; cellulitis/abscesses; mandibular fractures; temporomandibular joint dislocation; dento-alveolar traumas (dislocations, avulsions, dental fractures with the impairment of the pulp chamber); and ulceronecrotic gingivostomatitis.

Dental clinics were suspected to be a hotspot for nosocomial transmission of COVID-19 due to the easy spread of the virus [4]. Dentistry was declared one of the most exposed professions to the COVID-19 contagion. Studies proved that blood droplets and saliva that are deposited on the metal surfaces or aerosol inhalation generated by rotating instruments and ultrasound hand pieces put in high risk both health professionals working in dentistry services and patients [5,6,7].

Many studies proved that COVID-19 transmission occurs through four major routes: (1) direct exposure to respiratory secretions containing droplets, blood, saliva, or other patient materials; (2) indirect contact with contaminated surfaces and/or instruments; (3) inhalation of suspending airborne viruses; and (4) mucosal (nasal, oral, and conjunctival) contact with infection-containing droplets and aerosols that are propelled by coughing and talking without a mask [8]. Thus, the coronavirus pandemic has brought an unprecedented new challenge to the health dentistry and that is why, in the beginning of pandemic, few studies succeed to provide solid and rapid scientific data about the spread of COVID-19 infection in dental settings. Few available data were considered to have established guidelines promoting preventive behavior rules [9].

The new context of COVID-19 pandemic and the lack of solid scientific information had a negative impact on dentist practice and life. Most of the doctors who could work during the lockdown period (emergency departments, for example) followed the guidelines established by the professional societies and adjusted their rules by taking into consideration the specific context of their job (providing dental care to infected/non-infected/suspected/confirmed patients). Being a front-line dentist during the COVID-19 pandemic was a risky and challenging job. Apart from the need to apply specific dental methods to diminish the risk of spreading the infection (pre-procedural use of mouthwash, the use of rubber dam, and anti-retraction hand piece) dentists had to wear a fully protective suit, which was sometimes difficult to find. During the first months of the pandemic, the necessary medical supplies and COVID-19 protection equipment (hazmat, helmet, protective waterproof overalls with hood suitable for biohazard, single use surgical caps, disposable gowns, surgical masks, FFP3/FFP2 masks, face shields, goggles with side protection or glasses, gloves, single use shoes protection, single use examination gloves with long sleeves, single use reinforced surgical gowns) were missing all over the world.

In many countries, dentists were forced to stop working during lockdown until further notification. Self-isolation, the fear of infection, and the financial impact on family determined physical and psychological pressure. Studies conducted in UK, China, Israel, Romania, Mexico, Poland, Italy, and Spain showed that dentists were affected by depression, social anxiety, sleep-related problems, or new psychosomatic disorders were diagnosed during the first months of pandemic period. In a research including 30 countries, the authors showed that more than three quarters of the investigated dentists were anxious and scared by the devastating effects of COVID-19 [10,11,12,13,14,15,16]. For those continuing working in emergency units, a high level of stress was registered, determined especially by the fear of infection or the fear of transmitting the infection to their own family. That is why most of the dentists self-isolated from family members [17,18,19].

The only research in Romania focusing on dental emergency services during COVID-19 pandemic was conducted by Petrescu et al. [20] in one of the largest counties of Romania. The paper showed that the number of patients decreased, so, besides the economic and social life of the population, the pandemic impacted the use of medical care services, with important consequences during the post-lockdown period (an increased number of dental problems and patients). The main cause of attendance identified by the authors was acute apical periodontitis and acute pulpitis. But no other study investigated the changes in the personal, familial, and professional life of dentists in Romania and how the preventive behaviors were applied to diminish the risk for infection or fear of COVID-19.

The present paper is the first one focusing on preventive behaviors and the presence of fear of infection with COVID-19 among dentists in the Oradea region, Romania, during the first months of the pandemic. The primary objective of the research is to evaluate the level of fear of COVID-19 infection and identify preventive behaviors such as social distancing, washing hands, wearing masks and protective suits, using extra-care facilities in the office, applying new rules for patients’ triage and the use of supplementary methods to identify possible infected patients, avoiding large gatherings of people, or staying away from the family across samples of dentistry doctors in Romania.

## 2. Materials and Methods

### 2.1. Participants

This cross-sectional study was conducted in the Oradea area, a city situated at the north-western Romanian border. Being close to the country border, the region applied, from the beginning of the pandemic, very restrictive rules to diminish the level of infection brought by the migrants/travelers who entered Romania.

In November 2020, 457 dentists were registered at *Oradea College of Dentists*. Oradea is a university city with a Dentistry Department in the Faculty of Medicine and Pharmacy at the University of Oradea. The questionnaire was created using the free-access Google Forms application and the link to the online survey was sent to dentists working in public or private clinics. No incentives were offered to the respondents. The data was collected between 18 November and 5 December 2020, after seven months of the official beginning of the COVID-19 pandemic. The inclusion criteria were dentists registered at that time by the *Romanian College of Dentists*, working in public or private dental clinics or Dental Emergency Units. The exclusion criteria were age (employees after retirement age—65 years old), incomplete questionnaires (not fully filled in), and questionnaires returned after deadline.

### 2.2. Data Collection

A tool was constructed especially for the purpose of this research. The study collected information with regard to the following issues:

- Sociodemographic data (age, gender, length of employment, number of children, marital status, length of employment, environment, type of institutions, category of patients, level of specialization,) and medical-related data (chronic disease, previously infected with COVID 19).
- Job-related data in the first seven months of lockdown (number of working hours per week, number of free days, period of not working due to government restrictions, supplementary measures taken to diminish the risk of being infected with COVD-19, providing medical services only to dental emergency, etc.).
- Preventive behaviors adopted in the first seven months of confinement in the professional and private life (living separately from family members, medical suppliers, disinfection, schedule, pre-trial of patients, medical suits, etc.). Also, items investigated the institutional rules that changed to limit the spread of infections during dental procedures. Self-rated items collected answers using a Likert like scale with 5 points from 1 (*never*) to 5 (*always*).
- Self-rated item regarding the fear of being infected with COVID-19 and its consequences on health status. These statements were associated with a 5-point Likert scale from 1 (*never*) to 5 (*always*).
- Fear of COVID-19 developed by Ahorsu et al, in 2020 [21]. The tool included 7 items and respondents had to indicate their level of agreement with the statements using a five-item Likert-type scale. Answers included “strongly disagree”, “disagree”, “neither agree nor disagree”, “agree”, and “strongly agree”. The minimum score possible for each question is 1, and the maximum is 5. A total score is calculated by adding up each item score (ranging from 7 to 35). The higher the score, the greater the fear of COVID-19. This is an instrument that was frequently used in many scientific papers and proved to have a good reliability.

### 2.3. Statistical Analysis

All analyses were performed using *IBM* Statistical Package for Social Sciences (SPSS) Statistics for Windows, version 25 (SPSS Inc., Chicago, IL, USA). Results for descriptive statistics were expressed as means and standard deviations (SD), frequencies, and percentages. Mann–Whitney test was performed for the comparative analysis and for correlation analysis and the results were obtained using Spearman correlations. The level of statistical significance was set at *p* < 0.05.

### 2.4. Ethical Approval

The present study was conducted in accordance with the Declaration of Helsinki, and the protocol was approved by the Ethical Committee of Emergency County Hospital of Oradea, Romania, with the registration number No. 26214/18.November.2020. Before starting the survey, the participants were informed about the purpose of the research, the use of the results and confidentiality of data. Those who agreed to participate could fill in the questionnaires distributed online.

## 3. Results

### 3.1. Sociodemographic Data

The questionnaire was addressed to dentists working in public or private institutions from urban or rural areas and providing medical services for both children and/or adults. 

**Figure 1:**
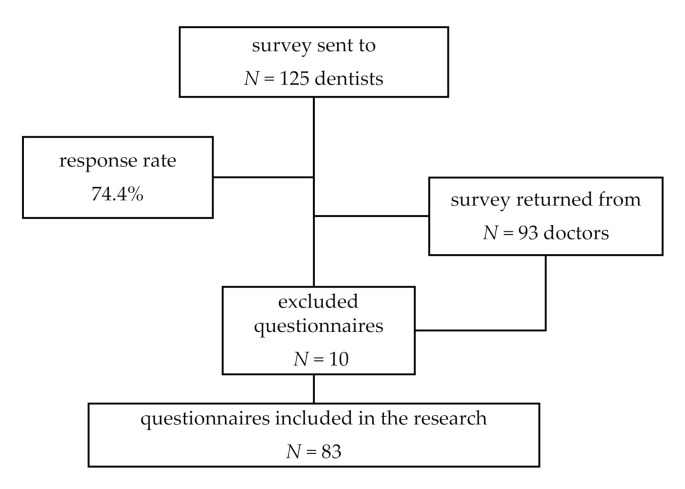
Study profile.

Figure 1 provides details on response rate.

Other sociodemographic data were collected (age, gender, length of employment, number of children, marital status, and teaching activity). The sociodemographic characteristics are presented in Table 1.

More than half of the respondents were parents (55.42%) of one child (*N* = 22, 26.51%), two (*N* = 19, 22.89%), three (*N* = 4, 4.82%) or four children (*N* = 1, 1.20%).

The experience in the dentistry field was 11.87 ± 8.62 years (with a minimum of 1 and a maximum of 24 employment years) and the number of working hours per week was 30.94 ± 11.76 (with a minimum of 4 and a maximum of 60 clinical hours) with differences due to the fact that university teachers usually have a small number of hours for clinical practice.

Only 9 doctors (10.84%) declared that they suffered from a chronic disease and all of them sustained that they were under medical treatment.

The number of dentists who were infected with COVID-19 in the seven months from the beginning of pandemic was small; 8 of the doctors (9.64%) were suspected of being infected and only 3 of them (3.61%) were confirmed as being positive. The percentages were higher in case of family members. During the investigated period, almost a quarter (*N* = 21, 25.30%) of the doctors had at least one member of the extended family confirmed with infection with COVID-19.

### 3.2. Financial Problems and Family-Related Data

In the first seven months of the pandemic period, almost half of the dentists (*N* = 37, 44.58%) had direct contact with patients suspected of being infected with COVID 19 and a quarter of them (*N* = 19, 22.89%) treated positive patients.

Dentistry is one of the medical specialties with a high risk of getting infected with COVID-19. Almost a quarter of the doctors (*N* = 20, 24.10%) declared that they have periods when they lived away from home being afraid of transmitting the disease to their family members. We found that that dentists who have children (M = 1.87) had multiple periods in which they did not live at home for fear of infecting family members (z = −2.609, *p* = 0.009), unlike dentists who were not parents (M = 1.62).

The COVID-19 pandemic impacted considerably dentists’ activity. Most of the doctors were not allowed to work. Many private and public clinics were closed in the first 2 months of the pandemic and only emergency units provided medical services to patients with specific diagnostics. More than half of the dentists (65.51%) declared that the number of patients decreased since the beginning of pandemic.

Dentists were asked if the restrictions that influenced negatively their activity had a negative impact on their financial status. The answers were rated on a Likert like scale from 1 (*never true*) to 5 (*always true*) and we obtained an M = 3.85 ± 1.26. The number of weeks in which they were unable to work due to the restrictions imposed was about 10.70 ± 14.20. The respondents working in rural area were more negatively influenced by the emergency rules (M = 5) compared to dentists working in urban areas (M = 4, z = −2.666, *p* = 0.008) from the financial point of view. In fact, all doctors (100%) providing services in clinics from rural areas declared that the pandemics had a strong negative financial impact. No other differences were identified (considering gender, marital status, professional level, etc.) regarding financial issues.

In Romania, the employees in public medical emergency services were not allowed to take free days/holidays during the emergency period. We identified that more than one third of the dentists (*N* = 27, 32.53%) had no free days in the first seven months of pandemics and the average of free days for all the questioned doctors was 12.92 ± 19.97. We found significant differences between doctors with and without children, in what the number of free days was concerned (z = −2.090, *p* = 0.037), meaning that dentists who were parents had more days off (M = 14.7) than those who did not have children (M = 10.16).

### 3.3. Fear of COVID-19

#### 3.3.1. Self-Rated Items

The fear of getting the disease and transmitting it to their family members determined the dentists to adopt protective measures. The present study wanted to investigate the opinion of dentists regarding the risk of infection with COVID-19 during the first seven months of pandemic. Due to the fact the there was little information about how the virus was spreading and its consequences on health, dentists seemed to worry about three important things: not to be infected by the patients, not to transmit the infection to their parents and not to pass the infection on to their family members.

One third of dentists (*N* = 33, 39.8%) mentioned that they treated exclusively specific urgent dental problems since the onset of the COVID-19 pandemic. Also, 11 dentists (13.3%) declared that they refused to provide medical assistance to some specific pathologies because of the fear of COVID-19 infection.

At the same time, results of the Mann–Whitney test (z = −2.681, *p* = 0.007) showed that dentists suffering from chronic diseases increased anxiety when following news and stories related to Covid-19 on TV, media, or social networks (M = 3.67), as opposed to the seemingly healthy ones (M = 2.35).

A series of items investigated the fear related to the infection with COVID-19. The answers were rated on a Likert like scale, from 1 (*never*) to 5 (*always*). The results are presented in Table 2.

Comparative analysis revealed that there was an increased level of fear about the fact that that patients do not tell the truth regarding their health status. We found that the fear was greater (z = −1.617, *p* = 0.046) among dentists who do not have children (M = 3.86), as opposed to those who are parents (M = 3.15).

#### 3.3.2. Fear of COVID-19 Scale

For the present research, for Fear of COVID-19 scale, Cronbach Alpha was 0.89, which proved a good internal consistency. The total score was 14.56 ± 6.90 (ranged from 7 to 35). No significant differences between different variables (such as gender, marital status, diagnostic of a chronic disease, setting of work, etc.) were registered, but correlational analysis showed that there were positive correlations between total score for FCV-19S and some variables which refer to dental procedures. We identified that the more the dentists were afraid of contamination, the more they would try to shorten the duration of dental interventions for fear of not staying in contact with patients for long (r = 0.488 **, *p* < 0.001). The fear of infection had a significant correlation with the belief that dental procedures were a major source of infection (r = 0.415 **, *p* < 0.001) and that the opinion that dentists have an increased risk of infection with the new virus in their patients (r = 0.598 **, *p* < 0.001).

Negative correlations were identified between total score and some demographic variables, and we identified that the more children the dentists had (r = −0.301 *, *p* = 0.007), the less intense was the fear of COVID-19. Also, the higher the score for fear of COVID-19 was, the less the number of consultations was (r = −0.381 **, *p* = 0.001).

The results showed that there was a positive significant correlation between the score for fear of COVID-19 and: the fear of getting infection from co-workers (r = 0.452 **, *p* < 0.001), the fear of infection while taking off the protective suit (r = 0.293 *, *p* = 0.009), or the fear of getting infected because patients cannot wear a mask during a medical treatment (r = 0.370 **, *p* = 0.001).

### 3.4. Patient’s Safety, Triage, and New Protective Measures

Doctors considered that there was a remarkably high risk of being infected from their patients in dental settings. When asked to rate the presence of a high risk to get the infection from the patients from 1 (*never*) to 5 (*always*), the result obtained was M = 3.51 ± 1.51. On the other hand, 66.27% (*N* = 55) sustained that they had never asked a patient to provide the proof of a negative test. Dentists were asked to rate from 1 (*never*) to 5 (*always*) some preventive measures that they applied in their dental clinics. Among the most frequent protective measures were mentioned: patients wear surgical masks while in the waiting room, patients disinfect their hands when entering the office, epidemiological triage was done by filling out a triage form by the patient, patients were measured temperature before providing medical care, patients had to wear protective equipment (disposable gown, cap, and shoe protection). Also, family members were no longer received in the medical room. The results are presented in Table 3.

Strong positive correlations were identified among some preventive measures. We identified that the more the dentists asked their patients to rinse mouths with disinfectants, the more they would also apply the following preventive measures such as performing telephone epidemiological triage (r = 0.467 **, *p* < 0.001), scheduling their consultations (r = 0.221 *, *p* = 0.045), not accepting family members or caregivers in their dental room (r = 0.249 *, *p* = 0.023), and the more they would provide protection equipment to their patients (r = 0.419 **, *p* < 0.001).

Comparative analysis showed that dentists working in public dental clinics (M = 2.67) appreciated in greater proportion (z = −2.905, *p* = 0.009) the fact that the number of patients increased, comparing to dentists working in private settings (M = 1.36). At the same time, results of the Mann–Whitney test (z = −2.122, *p* = 0.034) showed that public dentists were trying more to shorten the duration of dental interventions for fear of staying in contact with patients a longer period (M = 4) than dentists from private dental care (M = 2). In contrast, dentists from private clinics (M = 5), considered to a greater extend that their patients follow protective measures against infection with COVID-19 (z = −2.216, *p* = 0.027), compared to dentists working in public clinics (M = 3).

### 3.5. Institutional Preventive Rules

The pandemic put a great pressure on dental clinics, also. For many weeks, protective medical equipment was missing and not all the dentists had medical protective equipment. In some private dental clinics, the medical suits were missing, because new rules imposed that practitioners wear a disposable suit for each patient. Dentists were asked how they chose the criteria for their protective suit: the price or the quality. The answers reflected that more than half of the respondents (65.1%, *N* = 54) were more interested in the quality of protective suits when purchasing them.

Dentists were asked if they were trained about how to use protective equipment. A total of 51 dentists (61.4%) positively answered to this question. The comparative analysis showed no differences between dentists working in public/private areas or urban/rural environment.

The dentists were asked to rate-on a scale from 1 (*low*)–10 (*high*) if they considered that they took all the necessary measures for personal disinfection at the end of the working hours. The analysis of answers showed an M = 6.24 ± 2.22. More items regarding preventive measures and new institutional dental rules are presented in Table 4.

Tele-dentistry increased during the research period. Online or by phone consultations, along with telephonic triage were new implemented practices since the onset of the pandemic, aiming to diminish the frequency of direct contact with patients or to provide dental services during the State of Alarm period. The analysis of the answers showed that the number of dentists who never provided online or by phone consultation was lower (*N* = 11, 13.25%). All the other respondents sustained that they rarely (*N* = 6, 7.32%), sometimes (*N* = 29, 34.34%), frequently (*N* = 19, 22.89%), or always (*N* = 18, 21.69%) used online or by phone consultations for their patients prior scheduling an appointment. 

## 4. Discussion

The present study represents a descriptive analysis of dentist opinions and preventive practices along with the evaluation of level of fear of getting infected after the first seven months since the beginning of the state of emergency period due to the COVID-19 pandemic. The items of the questionnaire were developed after reviewing pertinent literature and international guidelines. The survey was designed in the Romanian language and comprised questions pertaining to socio-demographic characteristics, dentists’ attitudes and perceptions toward COVID-19, opinions about infection, and preventive measures applied in dentistry settings to control the spread of the virus. Moreover, the investigation was also focused on the psychological impact and changes on the everyday personal and familial life and dental practice; and the evaluation of the total score of fear of COVID-19.

Several studies conducted in the last year on the impact of the pandemic on different social or ethnic groups highlighted the fact that there are significant differences in these groups, in terms of physical and mental survival in the face of pandemic. For example, Gross et al. [22] showed that African Americans and Hispanics were bearing more of the brunt of the disease compared to their Caucasian counterparts. The idea that the pandemic can be worse for some ethnic and racial groups, determined the scientists to consider that there is a “pandemic within the pandemic” [23]. Bearing this idea in mind, we can consider that among doctors, some specialties were in the frontline of fighting COVID-19, while other medical specialties did not directly treat infected patients, or they did not treat them at all (patients were confirmed negative and then hospitalized). Dentistry was considered one of the most exposed medical specialties to infection, especially because patients do not wear a protective mask while being treated and dental interventions increase the risks of transmitting the infections (via air, saliva, and blood). Also, because a COVID-19 test was not mandatory in dental settings, dentists could treat asymptomatic patients. In this case, measuring body temperature or using oral disinfectants was not enough to combat transmission. That is why, to a large extent, the surveyed doctors considered that there was a high risk of infection in dentistry settings, and almost one fourth of the doctors declared that they had periods when they decided to stay away from home.

Our results pointed out some supplementary measures that dentists performed to prevent the spread of infection. We found that 66.27% (*N* = 55) sustained that they never asked a patient to provide the proof of a negative test, but they imposed some other rules (they measured patient’s body temperature, they did not accept caregivers, they provided protective suits, masks, hand and oral disinfectants to the patients, they performed phone triage and office-based epidemiologic triage by filling in a medical document).

We found that 65.51% of dentists registered a decreasing number of patients since the beginning of the pandemic, and especially routine dental appointments became considerably rarer. Our results were congruent with those obtained in northern regions of Italy, where 70% of practice owners complained about the substantial decrease in the number of patients per day [17], with a great impact on their financial balance. Cancellation of appointments were due to three reasons: (a) fear of providing medical care in the early days of pandemic, (b) the closure of clinics due to governmental ordinance, and (c) the patients’ fear to look for routine dental care.

Our results were similar to those obtained by other researchers who pointed out that not only their colleagues were afraid of the situation, but also their patients, who were probably aware of the risks in the dental office. Consolo et al. showed that 92.7% of the dentists in Italy reported cancellation directly from patients [17]. In a study conducted in Poland, the authors showed that 71.2% of the respondents suspended their activity, and 51.6% of the number of respondents, who continued their activity, did so for reasons of ethics and medical ontology [16]. In our study, the dentists who reported important anxiety regarding financial status were especially from rural areas, and 100% of the questioned doctors reported that COVID 19 restrictions had a strong negative impact on their incomes. The financial problems identified were not due only to the closure of dental offices during the first two months of the pandemic. Our study showed that the dentists invested more in protective materials, respected more the disinfection interval between patients, and preferred good quality for protective equipment. Therefore, the concerns were also related to their future activity that would increase costs and reduce profit.

We found no difference between genders, marital status, or professional level, related to financial worries. In opposition to our results, Chamorro-Petronacci et al. [18] found that financial repercussions seemed to be higher in male participants, revealing higher economic losses than females.

Data collected showed that 9.64% of the dentists were suspected of being infected, but 3.61% were confirmed as being positive. In Romania, all confirmations of COVID-19 were made by RT-PCR testing. Starting with January 2021, conformation was made with rapid antigen tests, which were performed only in special centers (in hospitals, public health departments, or private clinics accredited in this regard, which have an RT PCR tester). During the seven months of the pandemic (the period covered by the present study), 3,771,316 RT-PCR tests were performed (based on the case definition, on the medical protocol or on request).

We found also that higher percentages were registered among family members. More than a quarter (25.30%) of the doctors had at least one member of the extended family confirmed with infection with COVID-19. Our results were higher than those reported by Consolo et al. [17], who found that only 1.1% of the dentists contracted COVID-19, while 68.6% knew at least one person who was infected.

In order to prevent infection and transmission of COVID-19 infection, additional protection measures and the use of new materials are required. These are controlled by regulations and codes and have been quickly adopted in most countries affected by the pandemic. The following are mandatory: epidemiological triage both online and in the office, dam isolation, thermometry, disinfection and rigorous ventilation of spaces, additional protective equipment (PPE), etc. To be effective in combating the spread of SARS-COV-2, this equipment must first be handled correctly, secondly it must be of good quality and it must cover the daily requirements. Especially in the first period of the pandemic, when the sanitary systems were overloaded, medical protective equipment was difficult to procure, and its price was regulated rather by scarcity and not by its quality. Therefore, some of the dentists voluntarily reduced the number of patients in order to be able to cope correctly with the new rigors imposed by limiting the infection. In our study, more than 65% of respondents chose quality equipment, and 61% of them were taught how to use this equipment. These percentages were similar to those reported in other studies—64% of dentists surveyed in Italy were trained on how to properly use protective equipment within their institution).

Our study revealed that, in great proportion, dentists respected the guidelines imposed by the dentistry associations and the practice of preventive behaviors were not revealed of being influenced by the environment or type of institution. Dentists proved to be aware of the fact that following guidelines will limit the spread of infection and will protect both doctor and patients. Apart from new preventive behaviors, we found that tele-dentistry was implemented by many respondents. Tele-dentistry is considered now a promising field, allowing dentist to maintain recall visits without physical contact and can be kept as a post-COVID method especially for medical cases with no radiological recommendation [24].

Self-rated items focused on identifying anxiety and fear related to the infection with SARS-CoV-2. We identified that a quarter of the dentists (24.10%) were not at all concerned about getting the infection from their patients, 13.25% of the doctors were not afraid about the fact that patients were not declaring the truth about their health state and only 1.2% sustained that they did not always care about imposed preventive behaviors. Additionally, counselling protective measures to patients, using self-protective suits and installing new rules in dental offices proved that the questioned doctors respected the guidelines applied in the dentistry field. Interesting results were reported in other surveys conducted in Israel and Italy who showed that dentists’ responses to prevention measures proved more concern about personal protective equipment, disinfection, and sanitation procedures than for measures applied to patients [12,17].

The instrument constructed by Ahorsu et al. [21] was used worldwide in the first year of the pandemic. The scale is a unidimensional one that measures one’s fear levels of COVID-19. The scale versions were immediately validated for the use in different languages/countries: Italy [25], South Arabia [26], Israel [27], Turkey [28], United States of America [29], Spain [30], China [31], Japan [32], Colombia [33], Norway [34], Argentina [35], Brazil [36], and Greece [37], and proved good internal consistency, good concurrent validity, and acceptable construct validity. Cronbach’s alpha score was 0.89, proving a very good internal consistency for our research. The analysis revealed a score of 14.56 ± 6.90 for Fear of COVID 19 Scale, comparable with those mentioned in Italian dentists (15.03 ± 5.45) [38].

Mekhemar et al. [39] found that female dentists, aged 50–59, chronically ill, working at a dental practice, and considering the COVID-19 pandemic a financial hazard registered higher scores for depression, stress, and anxiety. As our study revealed, COVID-19 had a considerable psychological impact on dental practitioners. Congruent with our results, Anmar et al. [40] found a positive association between change in behaviors and worries, meaning that worries were increasing the practice of preventive behaviors among dentists. Our comparative analysis proved no differences between genders, group ages, or professional levels. As we expected, the score for fear of COVID-19 was correlated with the practice of preventive measures, meaning that the higher the score was, the more preventive measures were applied. Even if studies identified that the use of FFP2 masks was associated with headaches (47.5%), severe exertion and discomfort (50.8%), moderate concentration problems (54.3%), moderate breathing difficulties (63.5%), and impaired work ability (85.5%) as Farronato et al. [41,42] showed in their studies, protective behaviors were adopted by all dentists and considered fundamental in protecting dentists and their patients.

We found that dentists suffering from chronic diseases increased anxiety when following news and stories related to COVID-19 on TV, media, or social networks. This result was due to the negative impact that media proved to have on the mental health of populations from different countries. The increasing numbers of fatalities presented by the medical staff were registered among vulnerable patients like older persons or those with different chronic diseases (but not only because SARS-CoV-2 seemed to affect more some of them, but also because a lot of patients did not have access to proper medical services or delayed the medical consultation due to fear of infection). Thus, increased level of anxiety, depression, and stress among dentists were frequently reported [11,13,17].

### 4.1. Strengths and Limitations of the Study

This study is the first on its kind conducted in Romania. The results are important because they shape the context and measures applied during the first seven months of the COVID-19 pandemic. The results obtained are useful for institutions and professional associations, which could increase the number of online trainings or provide supplementary guidelines (most of them were recommendations based on evidence from similar diseases, such as SARS and MERS) under the new discoveries about the virus. Also, further studies could be done to shape comparative results.

The limitations of this study include the fact that this is a cross-sectional one that can only prove the association between variables and not a cause–effect relationship. Because our data were collected in a limited time, under a stressful condition consecutively with the State of Alarm, we assumed the possibility that the level of knowledge regarding disinfection and its guidelines will be lower comparative with studies conducted after one year since the beginning of pandemic. We accessed the knowledge and attitude of dental health practitioners after the first seven months, but we were not able to study the impact of dental management practices among dentists or on patients’ oral health. At the start of the present study, in Oradea Metropolitan Area, the incidence rate was 5.87‰. Therefore, we consider that, depending on the incidence rate, preventive behaviors could change under the pressure of policymakers [43]. Future studies should focus on the impact of preventive rules and behaviors into practice and on the quality of oral health among patients, which was not applicable in our case.

### 4.2. Reflections and Planning

Due to the high risk of nosocomial transmission and taking into account that every person, doctor, or patient is potentially contagious, all prevention measures established for dental settings must be applied to prevent direct and indirect transmission of the virus. Ideally, patients and dentists should be tested for COVID-19 prior the dental treatment, but the costs will be higher. Dentists and the administrators of public or private clinics should be aware that new variants of the virus demand stronger recommendations and new updates.

## 5. Conclusions

The COVID-19 pandemic affected both private and professional lives of the dentists in Romania. One third of dentists mentioned that they had treated exclusively specific urgent dental problems since the onset of the pandemic and more than ten percent sustained that they did not provide medical assistance to all dental pathologies. The results of the present study mirrored the new context, rules, and challenges that dentists were facing during the first seven months of the lockdown. Policymakers and professional associations may benefit from these findings while formulating guidelines to support dentists during COVID-19 or any future pandemics, as well as dentists that should be aware that psychological health, familial context, or financial balance are contextual consequences that must be regulated under the auspices of pandemics.

## Figures and Tables

**Table 1:** The distribution of respondents considering the gender and professional level ^1^.

Sociodemographic and Medical Characteristics	N (%)/M ± SD ^1^
Age	37.81 ± 8.45
Length of employment (years)	11.87 ± 8.62
Gender	
Male	29 (34.94)
Female	53 (63.86)
I prefer not to say	1 (1.20)
Marital status	
Single	11 (13.25)
In relationship	72 (86.75)
Having children	
yes	46 (55.42)
no	37 (44.58)
Level of specialization	
Dentists	38 (45.78)
Male	17 (20.48)
Female	21 (25.30)
Residents	16 (19.28)
Male	1 (1.20)
Female	15 (10.64)
Specialists	10 (12.05)
Male	6 (7.23)
Female	4 (4.82)
Consultants	19 (22.89)
Male	5 (6.02)
Female	13 (15.66)
I prefer not to say	1 (1.20)
Teaching activity	
yes	23 (27.71)
no	60 (72.29)
Type of institution	
only public sector	3 (3.61)
only private sector	50 (60.24)
both private and public sectors	30 (36.14)
Providing medical assistance to	
only children	0
only adults	5 (6.02)
both children and adults	78 (93.98)
Working environment	
urban	77 (92.77)
rural	6 (7.23)

^1^ Number of answers (*N*) and percentage (%), Means and standard deviations (M ± SD).

**Table 2:** Fear of infection—self-rated items.

Items	M ± SD ^1^
I fear that wearing the protective suit against COVID-19 does not protect me enough	2.55 ± 1.41
I fear that patients do not tell the truth about their health	3.47 ± 1.40
I fear that I can get infected when I take off my protective suit	2.51 ± 1.43
I fear that I might get infected from co-workers	2.54 ± 1.30
I believe that dentists have a very high risk of COVID-19 infection by their patients	3.52 ± 1.51
Dental procedures can be a source of infection and spread of COVID-19	2.81 ± 1.26
The fact that patients cannot wear a mask during medical treatment causes me to fear infection	2.48 ± 1.35
I fear I can get infected by my patients	2.78 ± 1.38
I think my patients are afraid of getting infected with COVID-19 after dental procedures	3.01 ± 1.37
I have noticed that patients avoid coming to routine dental check-ups	3.49 ± 1.31

^1^ Means and standard deviations (M ± SD).

**Table 3:** Supplementary measures and new practices adopted to diminish the risk of infection.

Items	M ± SD ^1^
As an additional measure, I no longer receive family members in the medical room	4.09 ± 1.29
I ask patients to get a COVID test before giving them medical care	1.51 ± 0.84
As an additional measure, I measure the patients’ temperature before providing them with medical care	4.54 ± 1.15
As an additional measure, patients disinfect their hands when entering the office	4.81 ± 0.49
As an additional measure, patients wear a surgical mask while in the waiting room	4.85 ± 0.56
As an additional measure, I offer patients protective equipment (disposable gown, cap and shoe protection)	4.18 ± 1.23
As an additional measure, patients should rinse their mouth with disinfectants for the oral cavity	4.14 ± 1.36
To avoid contact with blood or saliva I use modern isolation systems	3.60 ± 1.25
As an additional preventive measure, I schedule consultations	4.79 ± 0.71
As an additional measure, I perform telephone epidemiological triage	4.07 ± 1.26
As an additional measure, I perform the epidemiological triage by filling in a triage form by the patient	4.63 ± 0.82
Patients are scheduled according to the severity of the condition	3.84 ± 1.40
I try to shorten the duration of dental interventions for fear of not staying too long in contact with patients	2.75 ± 1.51
I change my protective suit after each patient	3.61 ± 1.35

^1^ Means and standard deviations (M ± SD).

**Table 4:** Preventive behaviors and institution rules.

Items	M ± SD ^1^
At my workplace, the additional protection measures against COVID-19 infection are effective	4.51 ± 0.78
My workplace provides me with the protection equipment against COVID-19 infection	4.14 ± 1.53
Since the onset of the pandemic, we have provided medical care to patients online or by telephone	3.32 ± 1.26
I had difficulty in purchasing myself a protective suit/mask/visor	2.61 ± 1.59
I respect the time interval for performing the disinfection	4.65 ± 0.63
I keep the social distance from the auxiliary staff	4.12 ± 1.17
I fully comply with the hygienic-sanitary measures to prevent COVID-19 infection imposed by the competent authorities	3.81 ± 1.40

^1^ Means and standard deviations (M ± SD).

## Data Availability

The data presented in this study are available on request from the corresponding author.
